# Development of a fingerprinting panel using medically relevant polymorphisms

**DOI:** 10.1186/1755-8794-2-17

**Published:** 2009-04-20

**Authors:** Deanna S Cross, Lynn C Ivacic, Catherine A McCarty

**Affiliations:** 1 Center for Human Genetics, Marshfield Clinic Research Foundation, 1000 N. Oak Avenue, Marshfield, WI, USA

## Abstract

**Background:**

For population based biorepositories to be of use, rigorous quality control and assurance must be maintained. We have designed and validated a panel of polymorphisms for individual sample identification consisting of 36 common polymorphisms that have been implicated in a wide range of diseases and an additional sex marker. This panel uniquely identifies our biorepository of approximately 20,000 samples and would continue to uniquely identify samples in biorepositories of over 100 million samples.

**Methods:**

A panel of polymorphisms associated with at least one disease state in multiple populations was constructed using a cut-off of 0.20 or greater confirmed minor allele frequency in a European Caucasian population. The fingerprinting assay was tested using the MALDI-TOF mass spectrometry method of allele determination on a Sequenom platform with a panel of 28 Caucasian HapMap samples; the results were compared with known genotypes to ensure accuracy. The frequencies of the alleles were compared to the expected frequencies from dbSNP and any genotype that did not achieve Hardy Weinberg equilibrium was excluded from the final assay.

**Results:**

The final assay consisted of the AMG sex marker and 36 medically relevant polymorphisms with representation on each chromosome, encompassing polymorphisms on both the Illumina 550K bead array and the Affymetrix 6.0 chip (with over a million polymorphisms) platform. The validated assay has a P(ID) of 6.132 × 10^-15 ^and a P_sib_(ID) of 3.077 × 10^-8^. This assay allows unique identification of our biorepository of 20,000 individuals as well and ensures that as we continue to recruit individuals they can be uniquely fingerprinted. In addition, diseases such as cancer, heart disease diabetes, obesity, and respiratory disease are well represented in the fingerprinting assay.

**Conclusion:**

The polymorphisms in this panel are currently represented on a number of common genotyping platforms making QA/QC flexible enough to accommodate a large number of studies. In addition, this panel can serve as a resource for investigators who are interested in the effects of disease in a population, particularly for common diseases.

## Background

With the advent of genome wide association studies large bio-banks will become the stepping stones to tomorrow's medicine. The genome wide association study (GWAS) has recently proved its value with replicable findings for diseases such as coronary artery disease, prostate cancer, and diabetes [1], and many more discoveries are expected in the near future. To determine these associations, studies have relied on large populations of well characterized cases and controls often combining several study populations in order to approach statistical significance [1]. Once these associations have been found in well characterized target cohorts, the findings must be replicated in a population based cohort before translation into clinical practice [2]. These cohorts will come from the large population based cohorts being collected in places like the Marshfield Clinic [3] and Vanderbuilt [4] and Kaiser Permenente [5].

For these biorepositories to be of use rigorous quality control and assurance must be maintained to minimize sample identification errors. The quality of the nation's biobanks is such a priority that the National Cancer Institute has established an Office of Biorepositories and Biospecimen Research with the specific mission to develop standards and determine the best practices and protocols for the burgeoning field [5]. Erroneous sample identification and mistaken sample handling can stay hidden for many years compromising results; this problem has plagued researchers that work with cell culture for years [6,7].

One strategy for reducing errors in sample identification is to increase the automation of sample acquisition, storage and handling. The PMRP repository used a semi-automated system of sample handling for recruitment in 2001 which included the Marshfield Clinic's practice management and laboratory information systems for subject recruitment and sample collection. PMRP currently uses the Nautilus^® ^(Thermo Fischer, PA, USA) biospecimen tracking system with an integrated plate reader for tracking of sample storage [8]. Other larger biorepositories such as the UK biobank have chosen to automate sample collection and storage to a larger degree in order to further reduce the possibility of incorrect sample identification [9]. While automation does rule out some possible sample identification mistakes it does not eliminate all potential error from the process as technicians are still involved in sample acquisition and processing. In addition, automation does not inform the quality of the specimen to help research personnel determine the usefulness of the sample nor does it provide a process for correcting and identifying errors that have occurred during previous sample collections. Even when samples are collected, processed, and aliquotted with minimal human handling, results from collaborating institutions must be checked to ensure no identification errors occurred in transferring either the samples or the data between institutions. Therefore it is imperative that an initial characterization of a sample includes both unique identifiers and measures of quality. This is particularly true when genotypic and phenotypic information are being combined in large association studies.

One method of identification would be to rely on markers developed for forensic applications. A number of different SNP panels have been developed to identify subjects; these panels range from a 21 SNP multiplex to a 52 SNP multiplex that has been validated in different populations [10-13]. However, the use of forensic panels for sample identification may create ethical and legal challenges. Increasingly biorepositories have also chosen to create their own SNP panels for quality control and assurance. The Wellcome Trust used a 38 SNP panel for quality control of the samples used for the genome wide association study of seven common diseases [14]. While any of these panels could be used for sample identification and tracking, they are not useful in characterizing the population in relation to polymorphisms already known to be associated with disease.

In an effort to balance the needs of quality control and genomic research we have designed and validated a panel of polymorphisms for individual sample identification that have been implicated in a wide range of diseases. In this paper we describe our process for designing the panel as well as the ability of this panel to uniquely discriminate samples using known allele frequencies. We also demonstrate how this panel may be used in the future by researchers investigating common diseases such as heart disease and obesity.

## Methods

### SNP panel design

The inclusion criteria for a polymorphism to be considered for use in the fingerprinting assay were as follows. First the polymorphism must have a confirmed minor allele frequency of approximately 0.20 or greater in a European Caucasian population, this allele frequency was chosen based on a theoretical assay of 34 polymorphisms, an average allele frequency of 0.20 was needed to uniquely identify 20,000 subjects. Second, the polymorphism it must be medically relevant; defined as having been associated with at least one disease state in multiple populations. Finally, the polymorphism must be easily detectable using the Sequenom platform (Sequenom, San Diego, CA, USA).

In order to obtain an initial list of potential polymorphisms all of the investigators within the Marshfield Clinic Research Foundation were asked to submit polymorphisms that were of interest. Garnering a list of 116 polymorphisms, these were tested with the above criteria before proceeding to assay design. In addition, this list was supplemented with polymorphisms from the PharmGKB [15] and Genetic Association Database [16].

The minor allele frequency in a Caucasian population was then confirmed using the published allele frequencies in dbSNP [17] to ensure that the polymorphism was common enough to be considered for inclusion in the panel. A Medline [18] search for disease associations was then performed for each polymorphism that met the frequency requirement. Any polymorphism that had an association with at least one disease in two separate preferably Caucasian populations was then allowed to progress to the next step.

Finally each polymorphism was masked using SNPmasker and evaluated for ease of adaptation to the Sequenom platform. The remaining polymorphisms were used in the Sequenom Assay Design 3.1 [19] program to develop a large multiplex assay. Several assays with between 33–39 polymorphisms as well as the sex marker AMG were created. A single assay of 40 total polymorphisms with at least one polymorphism on each chromosome was chosen for validation.

This study was reviewed and approved by the Marshfield Clinic Institutional Review Board.

### Allele detection

Allele detection was carried out using the MALDI-TOF mass spectrometry method of allele determination on a Sequenom platform. Briefly this method of detection depends on the different molecular weights of the polymorphic regions to determine the allele. A multiplexed PCR reaction is carried out on genomic DNA to amplify regions of interest. These products are then annealed with primers that are in the region directly adjacent to the polymorphism and a single base pair primer extension reaction is performed to generate the allele specific products. The products are then placed on a proprietary chip and analyzed using MALDI-TOF mass spectrometry and Sequenom Typer 3.4 software [20] to make the allele determination. A list of the PCR and detection primers is provided in additional file 1. Any of the automatic genotype determinations that were flagged as lower confidence or that the program failed to automatically determine were then double checked by a human to determine if manual allele calls could be made.

### Assay validation

The 40 polymorphism fingerprinting assay was tested initially on a panel of 28 Caucasian HapMap samples from the CEPH population provided by Coriell. Our fingerprinting assay contained 32 polymorphisms that had been previously genotyped in this population; we compared our results with those released through the dbSNP Genotyping detail [21] or the cancer500 web site [22] to ensure genotyping accuracy. Further validation for accuracy and precision was completed by repeated assays using anonymous DNA extracted with our protocol from a Caucasian population. One final population of a further set of 21 CEPH samples was used to ensure accuracy of both the automated and manual genotype calls. Polymorphisms that were not automatically determined at least 80% of the time were excluded from the assay. The frequencies of the alleles and genotypes obtained with the assay were compared to the expected frequencies from dbSNP. Finally, the polymorphisms in the assay were checked for disagreement with Hardy Weinberg equilibrium; any polymorphism that failed was excluded from the final assay.

### Determination of individual discriminatory power

The expected allele frequencies were used to determine the potential power of the assay using the following equations for the probability of identity. We calculated the probability of identity [P(ID)] for the assay using two different equations. The first equation assumes the population is random and uses the product rule with Hardy-Weinberg equilibrium assumptions [23], and the second equation is more conservative and assumes the population is composed of siblings [24]. We performed these calculations assuming independent assortment of the alleles.

(1)

(2)

## Results

### Polymorphism selection and assay design

**Figure 1:**
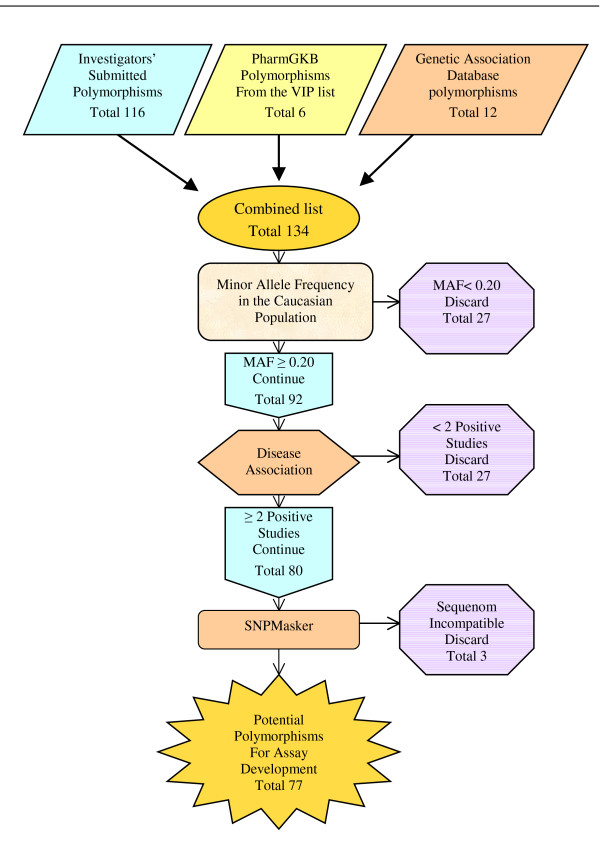
**Schematic representation of the development the DNA fingerprinting assay**. Schematic representation of the inclusion criteria for a polymorphism to be considered for the fingerprinting assay and the number of polymorphisms that failed each step.

116 polymorphisms were submitted by investigators from the Marshfield Clinic Research Foundation, 6 polymorphisms were taken from the PharmGKB VIP list and polymorphisms in chromosome 9, 18, 21, and 22 were obtained from the Disease Association Database providing an additional 12 polymorphisms. All of these polymorphisms were then evaluated for the inclusion criteria (Figure 1). 27 candidates failed because the allele frequency in a Caucasian population was below 20%. Another 21 polymorphisms failed to pass the criteria of medical relevance. Finally, three more polymorphisms were eliminated after masking because of incompatibility with the Sequenom system. The remaining 77 polymorphisms were placed in the Sequenom Assay Design program and a single 40 polymorphism assay was created. The assay includes 39 polymorphisms that are medically relevant (see additional file 2 for the references used to determine medical relevance) with at least one polymorphism on each chromosome and the AMG gender marker. The polymorphisms used in the assay as well as the chromosome and associated gene are provided along with at least one associated disease state (Table 1).

**Table 1:** Initial design of unique fingerprint assay using medically relevant polymorphisms

**Polymorphism**	**Caucasian MAF**	**Chromosome**	**Gene**	Disease Associated with Polymorphism *
rs1137101	0.449	1p31	LEPR	Obesity, Insulin Resistance, Non-Hodgkin's lymphoma

rs486907	0.408	1q25	RNASEL	Prostate cancer

rs1042031	0.208	2p24	APOB	Cardiovascular disease, Dislipidemia

rs231775	0.379	2q33	CTLA4	Multiple Sclerosis, Autoimmune Disease

rs5186	0.348	3q21	AGTR1	Metabolic syndrome, Aortic aneurism, Left-ventricular hypertrophy

rs6280	0.35	3q13.3	DRD3	Schizophrenia

rs1693482	0.477	4q21	ADH1C	Alcohol dependence, Coronary heart disease

rs1799883	0.373	4q28	FABP2	Metabolic syndrome, Type 2 diabetes

rs4444903	0.392	4q25	EGF	Cancer

rs4961	0.208	4p16.3	ADD1	Hypertension, Coronary artery disease

rs1042714	0.467	5q31	ADRB2	Obesity, COPD

rs351855	0.283	5q35.1	FGFR4	Cancer

rs5370	0.242	6p24	EDN1	Asthma, sleep apnea

rs6296	0.322	6q13	HTR1B	substance abuse

rs2227983	0.25	7p12.3	EGFR	Cancer

rs213950	0.492	7q31.2	CFTR	Cystic fibrosis

rs7493	0.237	7q21.3	PON2	Myocardial infarction

rs328	0.273	8p22	LPL	Left ventricular hypertrophy

rs2383206	0.475	9p21		Coronary artery disease

rs1800861	0.25	10q11.2	RET	Hirschsprung disease, Thyroid cancer

rs1801253	0.283	10q24	ADRB1	Insulin Resistance

rs2227564	0.341	10q24	PLAU	Alzheimer's disease, asthma

rs1799750	0.433	11q22.3	MMP1	Endometriosis, Osteolysis, Rheumatoid authritis

rs1063856	0.342	12p13.3	VWF	Hypertension

rs6313	0.438	13q14	HTR2A	Psychiatric disorders

rs2236225	0.396	14q24	MTHFD1	Neural tube defects

rs1800588	0.333	15q21	LIPC	Coronary artery disease

rs243865	0.198	16q13	MMP2	Cancer

rs4673	0.342	16q24	CYBA	Coronary artery disease

rs708272	0.478	16q21	CETP	Coronary artery disease

rs1800012	0.188	17q21.3	COL1A1	Osteoporosis

rs4291	0.354	17q23	ACE	Depression, Alzheimer's disease

rs4792311	0.331	17p11	ELAC2	Prostate cancer

rs16430	0.37	18p11.3	ENOSF1/TYMS	Cancer

rs601338	0.391	19q13.3	FUT2	Infection susceptibility

rs688	0.45	19p13.2	LDLR	Alzheimer's disease, Coronary artery disease

rs7121	0.458	20q13.3	GNAS	Obesity, Cancer

rs234706	0.333	21q22	CBS	Oral cleft defects

rs4680	0.483	22q11.21	COMT	Schizophrenia, ADHD

AMG (del)	0.5	Xp22.3	AMG	Sex Marker

Polymorphisms (rs1693482, rs4444903, rs1800012) were excluded from the final assay because of validation failures.

* Annotated bibliography of the publications used to determine medical relevance is in additional file 2.

### Assay validation

A total of 3 polymorphisms (rs1693782, rs4444903, and rs1800012) did not achieve 80% automated call rate and accuracy in the assay. The finalized validated assay consists of 37 polymorphisms, with the AMG sex marker and 36 medically relevant polymorphisms with representation on each chromosome (Table 1).

Since one of the goals of this assay is to provide a quality control mechanism for genotyping using a whole genome scan we assessed the panel for polymorphisms that were represented on either the Affymetrix 6.0 chip (with over a million polymorphisms) or the Illumina 550K bead set (with over half a million polymorphisms). The fingerprint encompasses 16 polymorphisms that are on the Illumina 550K assay and 12 that are on the Affymetrix 6.0 chip assay with 7 polymorphisms in common to both platforms (Table 2).

**Table 2:** Fingerprinting polymorphisms on either the Illumina or Affymetrix platform

**Polymorphism**	**Illumina Platform** **550K bead set**	**Affymetrix Platform** **6.0 chip set**
rs1137101	X	

rs486907	X	

rs1042031	X	

rs6280	X	

rs5370	X	

rs6296		X

rs213950	X	

rs7493		X

rs2227564	X	X

rs1063856	X	

rs6313	X	X

rs2236225	X	X

rs1800588	X	

rs243865		X

rs4673	X	

rs4792311	X	X

rs688	X	X

rs7121		X

rs234706		X

rs4680	X	X

AMG (del)	X	X

TOTAL	16	12

### Power of discrimination

Preliminary analysis of a hypothetical SNP panel suggested that 34 polymorphisms of an average minor allele frequency of 0.20 along with a sex marker would be needed to individually identify a biorepositiory of 20,000 samples. Here we have created a panel of 36 polymorphisms with an average MAF of 0.354667 and a sex marker. For the validated assay we obtained a P(ID) of 6.132 × 10^-15 ^and a P_sib_(ID) of 3.077 × 10^-8^.

To further ensure the assay could be used to uniquely identify our population we calculated the P(ID) and P_sib_(ID) of the assay assuming that only polymorphisms located farther than 50 megabases apart segregated independently. Finally we calculated the probability of identity using only the polymorphisms found on the Illumina and Affymetrix platforms, using only the polymorphisms limited to one or the other of these platforms individual identification of samples should be possible. Even using the most conservative P_sib_(ID) from the polymorphisms on the Affymetrix platform, 3.0 × 10^-3^, meaning 60 individuals in the population may not have unique patterns (Table 3).

**Table 3:** Probability of identity for the validated fingerprinting assay

**Polymorphism group**	**Total number of Polymorphisms**	**Probability of identity P(ID)**	**Probability of identity (siblings) P_sib_(ID)**
Validated fingerprinting assay	37	6.132 × 10^-15^	3.077 × 10^-08^

Conservative validated fingerprinting assay*	32	4.906 × 10^-13^	2.947 × 10^-07^

Polymorphisms on the Illumina platform (550K)	16	5.844 × 10^-07^	4.075 × 10^-04^

Polymorphisms on the Affymetrix platform (6.0 chip)	12	2.649 × 10^-05^	3.004 × 10^-03^

* The conservative estimate includes only polymorphisms that are greater than 50 centimorgans (cM) apart to ensure independent assortment of each polymorphism.

### Common Disease Coverage

The second goal of the fingerprinting panel is to provide a resource for investigating the genetic components of common diseases. In order to ensure that the fingerprint assay developed here encompasses a wide range of diseases we compared the coverage of the assay with the 28 focus areas from healthy people 2010 [25]. Many of the focus areas such as cancer, heart disease, diabetes, obesity, and respiratory disease are well represented in the fingerprinting assay. Heart disease, the most prevalent disease in the PMRP population and a high priority disease for healthy people 2010 is represented with 10 polymorphisms that may be important in the development or severity of disease (table 4). Based on the prevalence of these diseases in the population, the minimal detectable odds ratio for these polymorphisms is between 1.5 and 3.2 with the most prevalent disease (heart disease) having the lowest detectable odds ratio of 1.5 [26] (table 4).

**Table 4:** Common diseases in the PMRP cohort and polymorphism coverage of these diseases.

**Disease**	PMRP prevalence (%) *	**Associated polymorphisms in the fingerprint**	**Healthy People 2010 Focus area**	Minimal detectable odds ratio based on disease prevalence*
Cardiovascular disease	7518 (41.2%)	10	12	1.5

Obesity	6927 (38%)	7	19	<3.2

COPD	6123 (33.6%)	2	24	<3.2

Arthritis	4814 (26.4%)	3	2	<3.2

Asthma	2186 (13.7%)	4	24	<3.2

Cancer (all sites)	2073 (11.4%)	8	3	<3.2

Diabetes	1734 (9.5%)	4	5	<3.2

*from McCarty, Mukesh, Giampetro and Wilke 2007 [24]

As would be expected from a collection of medically relevant polymorphisms many of the genes represented in the assay share common pathways or substrates. Examples of pathways with more than one represented gene include the renin-angiotensinogen pathway with three genes (ACE, AGTR1, EGF), and the cholesterol maintenance pathway with four genes in the assay (APOB, CETP, LDLR, LPL, and FABP2). In addition, neurotransmitter pathways are represented by four different genes; two serotonin receptors (COMT, DRD3) and two genes in the dopamine pathway (HTR2A, HTR1B). Furthermore three genes in the assay use folate as a substrate (MTHFD1, ENOSF1, CBS).

## Discussion

As we usher in the era of genomic medicine, population based biorepositories linked to growing medical records will become important for translating genetic discoveries into medical practice. For these repositories to be of use, stringent quality control and assurance mechanisms must be established. Furthermore these repositories become the ideal place to test genetic associations that have been discerned in case control cohorts to determine the disease burden in an unselected population. In this paper we described the development of a single assay of 36 medically relevant polymorphisms and a sex marker that will be used to uniquely identify samples in the Personalized Medicine Research Project cohort and simultaneously provide investigators with information on medically relevant polymorphisms for a wide array of diseases. Furthermore we determined which of these polymorphisms were present on the two most popular platforms used for whole genome association, Illumina and Affymetrix, to ensure the panel would be adequate for determining sample handling errors in shipping and genotyping.

Quality control and assurance of biorepositories involves an integrated program of both minimization of potential errors and testing for potential sample problems to quickly rectify errors. The PMRP biorepository incorporates automation through an in house developed system for specimen acquisition and the Nautilis^® ^system for specimen tracking. However, automation is only one aspect of a comprehensive sample assurance program. This newly developed assay will be used for determining the ability of the DNA to be used for genotyping. In addition it will allow us to determine an estimate of error rates in our sample acquisition. We will compare the genotyped sex with the expected sex from the medical record to ensure a match. In addition, we will use known trios and pedigrees to compare individual samples for instances of non-maternity and non-paternity as potential indicators of sample identification error. Because of the unique ability of PMRP to re-contact subjects, as samples are exhausted or potential errors are identified, new samples can be acquired and verified using existing information to confirm sample identity. This ensures that each sample can be correctly attributed to an individual each time a new sample is drawn.

The number of polymorphisms used for our assay compares favourably to panels that have been used for other applications. In order to ensure that our goal of using the fingerprinting assay for individual identification of the PMRP cohort was met, we determined the P(ID) and P_sib_(ID) of the assay. Even using the most conservative estimates of the assay we can correctly identify approximately three million samples. SNP panels used for forensic applications have ranged from 20 to 52 SNPs [10,11,13]. A test of SNP panels used to identify cell lines included panels of 80, 60, 40, and 20 SNPs with the conclusion that 20 SNPs did not discriminate adequately however any of the most frequent 40 polymorphisms would provide adequate power of discrimination. From this assumption an assay of 34 polymorphisms with individual identification ability was developed [12].

Our assay is unique not only because we used only medically relevant polymorphisms but also because we developed the assay with a polymorphism on every chromosome and included polymorphisms on a number of popular genotyping platforms. Even using the most conservative estimate of P_sib_(ID) and only the polymorphisms on the Affymetrix platform we would have few potential incorrectly identified samples, and these samples could easily be further identified by ascertaining the genotype calls polymorphisms in linkage disequilibrium with polymorphisms that are on the fingerprinting assay. This ensures that any sample handling errors that are potentially introduced in shipping the samples or receiving data from other laboratories can be detected when genotyping results are entered into our database. By comparing returned genotypes with previously determined genotypes provides a quality check for any data that is generated externally. Using the assay in this manner provides an opportunity to correct sample errors or questionable calls from genotyping before samples are linked with phenotypic medical information.

Because of the dual nature of the assay several potential limitations arise. The stringent dual criteria of medical relevance and high minor allele frequency have the potential to limit the usefulness of the assay for both goals. One of the limitations of the assay we developed is that only the European Caucasian MAF was considered as a selection criterion. The PMRP biospecimens are overwhelmingly Caucasian therefore this is appropriate for our application, however this emphasis may decrease the applicability of this assay to other populations. Using the allele frequencies for Asian and African populations from dbSNP 6 polymorphisms in the assay fell below 10% MAF in Africans and 2 polymorphisms were below 10% in Asian populations. Identification panels developed for forensic applications generally consider a large number of different ethnicities when creating the panel to ensure correct identifications across populations [10,11,13]. At this time, we have no reason to believe that the choice of medically relevant polymorphisms inherently biased the assay toward the Caucasian population and preliminary analysis of estimated allele frequencies in other populations would suggest the fingerprinting assay to be robust for other populations. Myles et al. [27] recently tested allele frequencies across populations for disease associated SNPs and determined they vary no more across populations than randomly chosen SNPs. However any polymorphism can have varying allele frequencies among different populations and until the allele frequencies for our polymorphisms are verified in other populations we cannot determine how useful it would be for non-Caucasians.

Another limitation for the assay is that the medical relevance of the assay could be limited. Many medically relevant polymorphisms are less frequent in the population than 20%. For instance polymorphisms in the ApoE alleles are highly medically relevant with many pleiotropic effects on disease [28], however they have a minor allele frequency of 0.18 and 0.02 [21], and therefore were excluded from consideration. In addition, because of the evolving nature of gene disease discovery it is possible that some of the polymorphisms we included in the assay will later be determined to have little medical value. For instance, the polymorphism chosen in the RET gene while associated with disease, has only been associated in small studies [29,30]. Furthermore, newly associated medically relevant polymorphisms such as the cancer susceptibility locus on chromosome 8q24 [31] were not included in the assay because assay construction began before this region was known. Finally, because we focused on including polymorphisms from a range of diseases, it is likely that investigators will need to perform more genotyping for any comprehensive study of disease-gene interactions.

By using a limited number of commonly occurring polymorphisms as an identification panel and maintaining strict access to individual genotyping results, the panel avoids the potential legal and ethical burden of using a forensic panel, does not expose the population to increased identification risk, and may provide insight into population wide allele frequencies. As with any population wide genotyping, the manner of reporting the genotypes must be taken into consideration to minimize the risk of unintended identification. The genotypes created by this fingerprinting assay will be publicly reported only as population wide allele frequencies. Disease associated polymorphisms have been reported in this manner for a number of different populations, most recently the NHANES III population [32] and the CLUE I and CLUEII populations [33]. Even with the recent revelations regarding the ability to discern individual DNA contribution to a mixture, the number of potentially reported polymorphisms from this assay, 36, is well under the smallest estimate of 10,000 needed to discern an individual in a mixture of 0.1%[34]. Individual genotypes are stored in a secure server and not publically available. The PMRP consent form and study guidelines require any investigator that requests individual genomic information for any reason to have an IRB approved protocol before data is released [3].

Despite the potential limitations of the panel, we have demonstrated that an identification panel can be created using only medically relevant polymorphisms. Such panels could be created with disease specific polymorphisms and used in the future not only to identify samples but potentially to access an individual's genetic risk of a disease We are currently using this panel for QA/QC purposes on the DNA samples in the PMRP repository. In the future we intend to test these polymorphisms for association with disease in the entire PMRP population to determine their medical relevance in our population. In the future this panel or other rationally designed panels can be used on large populations not only as a quality control measure but also to begin to translate the results from the large genome wide studies into clinical practice. Because this panel includes polymorphisms that are relevant in a wide range of diseases the information gained from genotyping the entire PMRP population will be used to help facilitate population based research.

## Conclusion

We created a single panel of 36 somatic polymorphisms and a sex marker with the ability to discriminate individuals in a large biorepository (of potentially over 100,000,000 samples) using only common medically relevant polymorphisms. These polymorphisms are currently represented on a number of common genotyping platforms making QA/QC flexible enough to accommodate a large number of studies. In addition this panel can serve as a resource for investigators who are interested in the effects of disease in a population particularly for common diseases.

## Competing interests

The authors declare that they have no competing interests.

## Authors' contributions

DC – Was responsible for determining polymorphism allele frequencies, determining polymorphism medical relevance, supervising the project, approving the assay design, and writing the manuscript. LI – Was responsible for designing the assay, Sequenom implementation, and validation. CM – Was responsible for project conception, advice for project implementation. All authors have read and approved the final manuscript.

## Pre-publication history

The pre-publication history for this paper can be accessed here:



## Supplementary Material

Additional file 1 **List of all primers used with the Sequenom assay**. Excel file of the PCR primers and extension primers used for the Sequenom fingerprinting assay. Click here for file

Additional file 2 **Annotated bibliography for all of the polymorphisms used in the fingerprinting assay**. Annotated bibliography used to determine medical relevance of the polymorphisms in the fingerprinting assay. Each polymorphism must be associated with disease in two independent populations. Click here for file
