# Supplementary material for: Development of a fingerprinting panel using medically relevant polymorphisms
Source: BMC Med Genomics. 2009 Apr 20;2:17. doi: 10.1186/1755-8794-2-17 (PMC2684120; doi:10.1186/1755-8794-2-17)
Supplement: Additional file 2 — Annotated bibliography for all of the polymorphisms used in the fingerprinting assay. Annotated bibliography used to determine medical relevance of the polymorphisms in the fingerprinting assay. Each polymorphism must be associated with disease in two independent populations. [file 1755-8794-2-17-S2.doc]

**Annotated bibliography for polymorphisms used in the fingerprinting assay.**

**Polymorphism Gene**

**rs1137101 LEPR**

1) Duarte SF, Francischetti EA, Genelhu VA, Cabello PH, Pimentel MM. **LEPR p.Q223R, beta3-AR p.W64R and LEP c.-2548G>A gene variants in obese Brazilian subjects**. *Genet Mol Res.* 2007, 6:1035-43. A case control study of 200 cases and 150 controls of mixed ethnicity including European-Caucasian. They found an association between LEPR rs1137101 (Q223R) and obesity O.R. 1.79.

2) Chiu KC, Chu A, Chuang LM, Saad MF. **Association of leptin receptor polymorphism with insulin resistance.** *Eur J Endocrinol.* 2004, 150:725-9. 67 healthy Caucasian subjects were recruited into a cross-sectional study to determine insulin sensitivity. The LEPR polymorphism rs1137101 (Q223R) was significantly associated with insulin resistance.

3) Skibola CF, Holly EA, Forrest MS, Hubbard A, Bracci PM, Skibola DR, Hegedus C, Smith MT. **Body mass index, leptin and leptin receptor polymorphisms, and non-hodgkin lymphoma.Cancer.** *Epidemiol Biomarkers Prev.* 2004, 13:779-86. A case control study of 725 cases and 1566 controls. An association between obesity and rs1137101 (Q223R) with Non Hodgkin’s Lymphoma was found that elevated the risk of Non Hodgkin’s Lymphoma 2 fold.

**rs486907 RNASEL**

1) Rennert H, Zeigler-Johnson CM, Addya K, Finley MJ, Walker AH, Spangler E, Leonard DG, Wein A, Malkowicz SB, Rebbeck TR. **Association of susceptibility alleles in ELAC2/HPC2, RNASEL/HPC1, and MSR1 with prostate cancer severity in European American and African American men*.*** *Cancer Epidemiol Biomarkers Prev.* 2005, 14:949-57. A case control association study for genetic association of prostate cancer in 888 European American and 131 African American cases and 473 European American and 163 African American controls. The RNASEL rs486907 (Arg462Gln) polymorphism was associated with low grade prostate cancer in European Americans with no family history of prostate cancer (OR1.5).

2) Krüger S, Silber AS, Engel C, Görgens H, Mangold E, Pagenstecher C, Holinski-Feder E, von Knebel Doeberitz M, Moeslein G, Dietmaier W, Stemmler S, Friedl W, Rüschoff J, Schackert HK; German Hereditary Non-Polyposis Colorectal Cancer Consortium. **Arg462Gln sequence variation in the prostate-cancer-susceptibility gene RNASEL and age of onset of hereditary non-polyposis colorectal cancer: a case-control study.** *Lancet Oncol.* 2005, 6:566-72. A case control study of 251 patients with hereditary non-polyposis colorectal cancer and 439 controls to investigate the role of RNASEL in cancer onset. RNASEL polymorphism rs486907 (Arg462Gln) Gln genotype was significantly associated with and earlier age of onset of first cancer (between 4 and 6 years) when compared to individuals with the Arg genotype.

**rs1042031 APOB**

1) Chiodini BD, Barlera S, Franzosi MG, Beceiro VL, Introna M, Tognoni G. **APO B gene polymorphisms and coronary artery disease: a meta-analysis.** *Atherosclerosis.* 2003, 167:355-66. A meta analysis of 14 studies with a total of 1721 cases and 1959 controls found an increased risk of CAD OR (pooled) 1.73 for carriers of the AA genotype of rs1042031 (ECORI) .

2) Benn M, Nordestgaard BG, Jensen JS, Tybjaerg-Hansen A. **Polymorphisms in apolipoprotein B and risk of ischemic stroke.** *J Clin Endocrinol Metab.* 2007, 92:3611-7. Epub 2007 Jun 26. A prospective study of 9157 men and women in Copenhagen City Heart Study. Individuals homozygous for ApoB polymorphism rs1042031 (E4154K) allele K exhibited a 3 to 5 fold decreased risk in ischemic cardiovascular disease and ischemic stroke.

**rs231775 CTLA4**

1) Heggarty S, Suppiah V, Silversides J, O'doherty C, Droogan A, McDonnell G, Hawkins S, Graham C, Vandenbroeck K.**CTLA4 gene polymorphisms and multiple sclerosis in Northern Ireland.** *J Neuroimmunol*. 2007, 187:187-91. A case control study involving 246 patients with relapsing-remitting multiple sclerosis, 84 patients with primary progressive disease and 158 healthy controls. The A allele of rs231775 (+49A/G) was associated with relapsing remitting disease (O.R> 1.36 for the heterozygote and 1.70 for the homozygote).

2) Juran BD, Atkinson EJ, Schlicht EM, Fridley BL, Petersen GM, Lazaridis KN. **Interacting alleles of the coinhibitory immunoreceptor genes cytotoxic T-lymphocyte antigen 4 and programmed cell-death influence risk and features of primary biliary cirrhosis.** *Hepatology*. 2008, 47:563-70. A case control study of 351 patients with primary biliary cirrhosis and 205 controls. The GG genotype was associted with AMA positivity (antimitochondrial antibody) among PBC patients.

**rs5186 AGTR1**

1) Jones GT, Thompson AR, van Bockxmeer FM, Hafez H, Cooper JA, Golledge J, Humphries SE, Norman PE, van Rij AM. **Angiotensin II type 1 receptor 1166C polymorphism is associated with abdominal aortic aneurysm in three independent cohorts.** *Arterioscler Thromb Vasc Biol.* 2008, 28:764-70. A combination of 3 case controlled cohorts for a total of 1226 patients with susceptibility to abdominal aortic aneurism and 1723 controls. The C allele of rs5186 (A1166C) was associated with disease (OR1.60).

2) Smilde TD, Zuurman MW, Hillege HL, van Veldhuisen DJ, van Gilst WH, van der Steege G, Voors AA, Kors JA, de Jong PE, Navis G. **Renal function dependent association of AGTR1 polymorphism (A1166C) and electrocardiographic left-ventricular hypertrophy.** *Am J Hypertens.* 2007, 20:1097-103. A cross sectional study of 8592 inhabitants of Groningen Netherlands. The rs5186 (A1166C) allele CC genotype was associated with LVH in the presence of decreased renal function.

3) Abdollahi MR, Lewis RM, Gaunt TR, Cumming DV, Rodriguez S, Rose-Zerilli M, Collins AR, Syddall HE, Howell WM, Cooper C, Godfrey KM, Cameron IT, Day IN. **Quantitated transcript haplotypes (QTH) of AGTR1, reduced abundance of mRNA haplotypes containing 1166C (rs5186:A>C), and relevance to metabolic syndrome traits.** *Hum Mutat.* 2007, 28:365-73. Metabolic syndrome traits were strongly associated with rs5186 (A1166C) which showed decreased abundance of AGTR1 transcript levels.

**rs6280 DRD3**

1) Talkowski ME, Mansour H, Chowdari KV, Wood J, Butler A, Varma PG, Prasad S, Semwal P, Bhatia T, Deshpande S, Devlin B, Thelma BK, Nimgaonkar VL**. Novel, replicated associations between dopamine D3 receptor gene polymorphisms and schizophrenia in two independent samples.** *Biol Psychiatry.* 2006, 60:570-7. Erratum in: *Biol Psychiatry*. 2008, 63:341-2. A case control study with familial trios. In the United States portion of the study 351 cases with 151 trios and 274 controls determined DRD3 allele rs6280(Ser9Gly) was associated with schizophrenia (O.R. 1.5).

2) Domínguez E, Loza MI, Padín F, Gesteira A, Paz E, Páramo M, Brenlla J, Pumar E, Iglesias F, Cibeira A, Castro M, Caruncho H, Carracedo A, Costas J. **Extensive linkage disequilibrium mapping at HTR2A and DRD3 for schizophrenia susceptibility genes in the Galician population.** *Schizophr Res.* 2007, 90:123-9. A case control study of 260 schizophrenic patients and 354 controls. Significant differences in DRD3 allele frequencies between cases and controls.

**rs1693482 ADH1C**

1) Kuo PH, Kalsi G, Prescott CA, Hodgkinson CA, Goldman D, van den Oord EJ, Alexander J, Jiang C, Sullivan PF, Patterson DG, Walsh D, Kendler KS, Riley BP. **Association of ADH and ALDH genes with alcohol dependence in the Irish Affected Sib Pair Study of alcohol dependence (IASPSAD) sample.** *Alcohol Clin Exp Res.* 2008, 32:785-95. In a case control study of 575 independent subjects with alcohol dependence and 575 controls rs1693482 (Ile271Gln) was associated with alcoholism.

2) Heidrich J, Wellmann J, Döring A, Illig T, Keil U. **Alcohol consumption, alcohol dehydrogenase and risk of coronary heart disease in the MONICA/KORA-Augsburg cohort 1994/1995-2002.** *Eur J Cardiovasc Prev Rehabil.* 2007, 14:769-74. A cohort study of 3703 patricipants in the MONICA/KORA study were genotyped for ADH1C rs1693482. Participants who drank more than 14g of alcohol a day and were either homozygous or heterozygous for the slow metabolizing allele had a 64% reduction in coronary heart disease risk.

**rs1799883 FABP2**

1) Galluzzi JR, Cupples LA, Otvos JD, Wilson PW, Schaefer EJ, Ordovas JM. **Association of the A/T54 polymorphism in the intestinal fatty acid binding protein with variations in plasma lipids in the Framingham Offspring Study.** *Atherosclerosis.* 2001, 159:417-24. A cohort study of 1930 participants showed an association between rs1799883 (A54T) and higher LDL cholesterol, and in women a significantly higher total cholesterol.

2) Fisher E,, Li Y, Burwinkel B, Kühr V, Hoffmann K, Möhlig M, Spranger J, Pfeiffer A, Boeing H, Schrezenmeir J, Döring F. **Preliminary evidence of FABP2 A54T polymorphism associated with reduced risk of type 2 diabetes and obesity in women from a German cohort.** *Horm Metab Res.* 2006, 38:341-5. A nested case control study of 192 cases of type 2 diabetes and 384 controls. The FABP2 homozygous T alleles were associated with a decreased risk of type 2 diabetes.

**rs4444903 EGF**

1) Lanuti M, Liu G, Goodwin JM, Zhai R, Fuchs BC, Asomaning K, Su L, Nishioka NS, Tanabe KK, Christiani DC. **A functional epidermal growth factor (EGF) polymorphism, EGF serum levels, and esophageal adenocarcinoma risk and outcome.** *Clin Cancer Res.* 2008, 14:3216-22. A case control study of 312 esophageal adenocarcinoma patients and 447 GERD free controls found that carriers of the rs4444903 (A61G) G/G alleles were at a 2 fold greater risk of developing esophageal adinocarcinoma (O.R. 1.81).

2) Tanabe KK, Lemoine A, Finkelstein DM, Kawasaki H, Fujii T, Chung RT, Lauwers GY, Kulu Y, Muzikansky A, Kuruppu D, Lanuti M, Goodwin JM, Azoulay D, Fuchs BC. **Epidermal growth factor gene functional polymorphism and the risk of hepatocellular carcinoma in patients with cirrhosis.** *JAMA*. 2008, 299:53-60. A case control study of 207 patients (Massachusetts General Hospital) with cirrhosis and a validation study with 121 patients (Hopital Paul Brousse) with cirrhosis were conducted. The rs4444903 (A61G) G/G genotype increased the odds of hepatocellular carcinoma by 4 fold (O.R. 4.0) in the original case control study and this was validated in the French cohort.

**rs4961 ADD1**

1) van Rijn MJ, Bos MJ, Yazdanpanah M, Isaacs A, Arias-Vásquez A, Koudstaal PJ, Hofman A, Witteman JC, van Duijn CM, Breteler MM. **Alpha-adducin polymorphism, atherosclerosis, and cardiovascular and cerebrovascular risk.** *Stroke.* 2006, 37:2930-4. A prospective study of 6471 subjects were genotyped for rs4961 (Gly460Trp). The variant allele was associated with atherosclerosis, cardiovascular disease, and cerebrovascular disease, with an increased risk in hypertensive subjects.

2) Related Articles, LinksPerticone F, Sciacqua A, Barlassina C, Del Vecchio L, Signorello MC, Dal Fiume C, Andreozzi F, Sesti G, Cusi D. **Gly460Trp alpha-adducin gene polymorphism and endothelial function in untreated hypertensive patients.** *J Hypertens.* 2007, 25:2234-9. Erratum in: *J Hypertens.* 2008, 26:380. The study included 110 Caucasian patients with chronic hypertension. Patients with at least one ADD1 (Gly460Trp, rs4961) Trp allele had impaired endothelium-dependant vaso-dilation (which is a predictor of cardiovascular risk).

**rs1042714 ADRB2**

1) Matheson MC, Ellis JA, Raven J, Johns DP, Walters EH, Abramson MJ. **Beta2-adrenergic receptor polymorphisms are associated with asthma and COPD in adults.** *J Hum Genet.* 2006, 51:943-51. A cross sectional population based study of 1,090 Caucasion participants. Homozygotes of the ADRB2 (Gln27Glu, rs1042714) Gln allele had an increased risk of asthma O.R. 2.08 and bronchial hyperactivity O.R. 1.92 and a haplotype including Gln27 and another ADRB2 polymorphism (Arg16) was associated with COPD O.R. 2.91.

2) Iaccarino G, Izzo R, Trimarco V, Cipolletta E, Lanni F, Sorriento D, Iovino GL, Rozza F, De Luca N, Priante O, Di Renzo G, Trimarco B. **Beta2-adrenergic receptor polymorphisms and treatment-induced regression of left ventricular hypertrophy in hypertension*.*** *Clin Pharmacol Ther*. 2006, 80:633-45. This study is a prospective follow up study of 970 hypertensive Caucasians. Carriers of at lest one Glu27 allele (rs1042714) presented with larger cardiac size (142.9 versus 138.2 p< 0.02). The Glu27 variant enhances hypertension-induced left ventricular hypertrophy.

3) Pereira AC, Floriano MS, Mota GF, Cunha RS, Herkenhoff FL, Mill JG, Krieger JE. **Beta2 adrenoceptor functional gene variants, obesity, and blood pressure level interactions in the general population.** *Hypertension.* 2003, 42:685-92. This study is a cross sectional study of 1576 individuals in Brazil (35% Caucasian). A 1.49 fold increase in obesity was found in individuals homozygous for the Gln27 variant of ADRB2 (rs 1042714).

**rs351855 FGFR4**

1) Spinola M, Leoni V, Pignatiello C, Conti B, Ravagnani F, Pastorino U, Dragani TA. **Functional FGFR4 Gly388Arg polymorphism predicts prognosis in lung adenocarcinoma patients.** *J Clin Oncol.* 2005, 23:7307-11. This study is a case control study of 274 lung cancer patients and 401 healthy control patients. Patients with at least one copy of the Arg388 allele (rs351855) exhibited an earlier age at cancer onset, higher proportion of poor clinical stage disease (HR 2.3), and increased nodal involvement (HR 1.9).

2) Thussbas C, Nahrig J, Streit S, Bange J, Kriner M, Kates R, Ulm K, Kiechle M, Hoefler H, Ullrich A, Harbeck N. **FGFR4 Arg388 allele is associated with resistance to adjuvant therapy in primary breast cancer.** *J Clin Oncol.* 2006, 24:3747-55. This is a study of 372 breast primary breast cancer patients. In node positive patients FGFR4 Arg388 allele (rs351855) was associated with poor disease free survival (p=0.02) and overall survival (p=0.04).

**rs5370 EDN1**

1) Zhu G, Carlsen K, Carlsen KH, Lenney W, Silverman M, Whyte MK, Hosking L, Helms P, Roses AD, Hay DW, Barnes MR, Anderson WH, Pillai SG. **Polymorphisms in the endothelin-1 (EDN1) are associated with asthma in two populations.** *Genes Immun.* 2008, 9:23-9. 342 families in the United Kingdom and 100 families in Norway were genotyped for 11 SNPs in EDN1. Polymorphism rs5370 was associated with asthma in both populations.

2) Diefenbach K, Kretschmer K, Bauer S, Malzahn U, Penzel T, Roots I, Fietze I. **Endothelin-1 Gene Variant Lys198Asn and Plasma Endothelin Level in Obstructive Sleep Apnea.** *Cardiology.* 2008, 112:62-68. This study is a case-control study of 364 consecutive patients with sleep apnea and 57 controls. The Lys198Asn allele (rs5370) was significantly associated with increased severity of sleep apnea especially in obese individuals.

**rs6296 HTR1B**

1) Quist JF, Barr CL, Schachar R, Roberts W, Malone M, Tannock R, Basile VS, Beitchman J, Kennedy JL. **The serotonin 5-HT1B receptor gene and attention deficit hyperactivity disorder**. *Mol Psychiatry*. 2003, 8:98-102. In 115 families the G allele of the HTR1B gene (rs6296) was found to be transmitted more often to the child affected with ADHD (p=0.09, chi^2^ =2.91).

2) Huang YY, Oquendo MA, Friedman JM, Greenhill LL, Brodsky B, Malone KM, Khait V, Mann JJ. **Substance abuse disorder and major depression are associated with the human 5-HT1B receptor gene (HTR1B) G861C polymorphism.** *Neuropsychopharmacology.* 2003, 28:163-9. 394 psychiatric patients and 96 controls were genotyped for rs6296 (G861C). Substance abuse (chi^2^ =9.51, p=0.009) and major depressive disorder (chi^2^ =6.83, p=0.033) were associated with the G681C allele.

**rs2227983 EGFR**

1) Press OA, Zhang W, Gordon MA, Yang D, Lurje G, Iqbal S, El-Khoueiry A, Lenz HJ. **Gender-related survival differences associated with EGFR polymorphisms in metastatic colon cancer.** *Cancer Res.* 2008, 68:3037-42. 318 patients with metastatic colon cancer were enrolled. In male patients the estimated survival time of for homozygous carriers of the Arg allele (rs2227983, R497K) had a decrease median survival time (10 months) while female homozygotes had an increased survival time (16 months) when compared to individuals of either sex with at least one lys allele (13.7 months) with a probability of gender interaction of p=0.003.

2) Wang WS, Chen PM, Chiou TJ, Liu JH, Lin JK, Lin TC, Wang HS, Su Y. **Epidermal growth factor receptor R497K polymorphism is a favorable prognostic factor for patients with colorectal carcinoma.**

*Clin Cancer Res.* 2007, 13:3597-604. 209 colorectal carcinoma patients were retrospectively analyzed. Of the 100 patients with stage II or stage III carcinoma Arg homozygotes (rs2227983, R497K) had decreased time of survival and overall survival (p<0.01).The Lys polymorphism was an independent predictor for survival.

**rs213950 CFTR**

1) Lee JH, Choi JH, Namkung W, Hanrahan JW, Chang J, Song SY, Park SW, Kim DS, Yoon JH, Suh Y, Jang IJ, Nam JH, Kim SJ, Cho MO, Lee JE, Kim KH, Lee MG. **A haplotype-based molecular analysis of CFTR mutations associated with respiratory and pancreatic diseases.** *Hum Mol Genet*. 2003, 12:2321-32. In 75 Korean patients with disseminated bronchietasis or chronic pancreatitis CFTR haplotype M470V (rs213950)-Q1352H was associated with bronchiectasis and chronic pancreatitis.

2) Ciminelli BM, Bonizzato A, Bombieri C, Pompei F, Gabaldo M, Ciccacci C, Begnini A, Holubova A, Zorzi P, Piskackova T, Macek M Jr, Castellani C, Modiano G, Pignatti PF**. Highly preferential association of NonF508del CF mutations with the M470 allele**. *J Cyst Fibros.* 2007 6:15-22. In 201 Italians and 73 Czechs with cystic fibrosis and their parents, the M allele of M470V (rs213950) was associated with carrying more potential cystic fibrosis mutations than the V allele. Carrying the M allele was associated with between a 6.9 and 15.4 percent increase in the chance of carrying a disease causing mutation in the CF gene.

**rs7493 PON2**

1) Guxens M, Tomás M, Elosua R, Aldasoro E, Segura A, Fiol M, Sala J, Vila J, Fullana M, Sentí M, Vega G, de la Rica M, Marrugat J; investigadores del estudio IBERICA**.[Association between paraoxonase-1 and paraoxonase-2 polymorphisms and the risk of acute myocardial infarction]** *Rev Esp Cardiol*. 2008, 61:269-75. A case control study of 746 acute myocardial infarction patients and 1796 controls from a Spanish population found that homozygous carriers of the S allele (C311S, rs7496) were at increased risk of acute myocardial infarction (OR 1.25).

2) Slowik A, Tomik B, Wolkow PP, Partyka D, Turaj W, Malecki MT, Pera J, Dziedzic T, Szczudlik A, Figlewicz DA. **Paraoxonase gene polymorphisms and sporadic ALS.** *Neurology.* 2006, 67:766-70. A case control study of a Polish population with 136 patients with large vessel disease (LVD) stroke, 140 with small vessel disease stroke, and 272 patients with cardioembolic stroke with age and gender matched controls. The PON2 allele (C311S, rs7493) was associated with LVD stroke (OR 1.58).

3) Slowik A, Wloch D, Szermer P, Wolkow P, Malecki M, Pera J, Turaj W, Dziedzic T, Klimkowicz-Mrowiec A, Kopec G, Figlewicz DA, Szczudlik A. **Paraoxonase 2 gene C311S polymorphism is associated with a risk of large vessel disease stroke in a Polish population.** *Cerebrovasc Dis*. 2007, 23:395-400. A case control study of 185 Polish patients with sALS diagnosis and 437 unrelated individuals. The C allele of the C311S allele (rs7493) was associated with ALS (OR 1.51) and when two polymorphisms analyzed together the R-C haplotype was associated with disease (OR 3.44).

**rs328 LPL**

1) Talmud PJ, Flavell DM, Alfakih K, Cooper JA, Balmforth AJ, Sivananthan M, Montgomery HE, Hall AS, Humphries SE. **The lipoprotein lipase gene serine 447 stop variant influences hypertension-induced left ventricular hypertrophy and risk of coronary heart disease.** *Clin Sci (Lond).* 2007, 112:617-24. A cohort study with 190 hypertensive individuals LPL-X447 allele (rs328) was associated with increased left ventricular mass P=0.01) but not in control subjects. In addition, in a cohort study of 2716 healthy men, hypertensive carriers had an increase risk of coronary heart disease (OR 1.54 to 2.3, P<0.0001).

2) Sass C, Herbeth B, Siest G, Visvikis S. **Lipoprotein lipase (C/G)447 polymorphism and blood pressure in the Stanislas Cohort.** *J Hypertens*. 2000, 18:1775-81. In a longitudinal cross sectional study of 767 mean and 816 women LPL (C/G) 447, G allele (rs328) was associated (p< 0.05) with lower pulse pressure and systolic pressure in women.

**rs2383206 9p21**

1) Abdullah KG, Li L, Shen GQ, Hu Y, Yang Y, Mackinlay KG, Topol EJ, Wang QK. **Four SNPS on Chromosome 9p21 Confer Risk to Premature, Familial CAD and MI in an American Caucasian Population (GeneQuest).** *Ann Hum Genet.* 2008 May 26 [Epub ahead of print]. A case control study using 310 cases of premature, famililial coronary artery disease (CAD) and 560 non –CAD controls (The GeneQuest population). All fours SNPs including rs238206 were associated with premature familial CAD.

2) McPherson R, Pertsemlidis A, Kavaslar N, Stewart A, Roberts R, Cox DR, Hinds DA, Pennacchio LA, Tybjaerg-Hansen A, Folsom AR, Boerwinkle E, Hobbs HH, Cohen JC. **A common allele on chromosome 9 associated with coronary heart disease**. *Science.* 2007, 316:1488-91. Six independent populations of more than 23,000 participants from four Caucasian populations were used. Individuals with the risk allele of rs2383206 were at 30-40% increased risk of CHD.

**rs1800861 RET**

1) Severskaia NV, Saenko VA, Il'in AA, Chebotareva IV, Rumiantsev PO, Isaev PA, Medvedev VS, Iasmita S. **[RET and GFRA1 germline polymorphisms in medullary thyroid cancer patients]** *Mol Biol (Mosk).* 2006, 40:425-35. This is a small case-control study of 67 medullary thyroid cancer patients and 178 ethnically matched controls. The L769L (rs1800861) variant was significantly decreased in cases versus controls.

2) Sangkhathat S, Kusafuka T, Chengkriwate P, Patrapinyokul S, Sangthong B, Fukuzawa M. **Mutations and polymorphisms of Hirschsprung disease candidate genes in Thai patients.** *J Hum Genet*. 2006, 51:1126-32. This is a small cohort study of 41 individuals of Asian decent who were diagnosed with Hirshsprung disease. The L769L allele (rs1800861), minor allele was statistically associated with long segment disease (OR3.462, p=0.021).

**rs1801253 ADRB1**

1) Mottagui-Tabar S, Hoffstedt J, Brookes AJ, Jiao H, Arner P, Dahlman I. **Association of ADRB1 and UCP3 gene polymorphisms with insulin sensitivity but not obesity**. *Horm Res.* 2008,69:31-6. A case control study of 292 obese and 481 non-obese females. ADRB1 polymorphism (rs1801253) was associated with serum insulin level (P=0.034) and insulin sensitivity (P=0.022).

2) Dionne IJ, Garant MJ, Nolan AA, Pollin TI, Lewis DG, Shuldiner AR, Poehlman ET. **Association between obesity and a polymorphism in the beta(1)-adrenoceptor gene (Gly389Arg ADRB1) in Caucasian women.** *Int J Obes Relat Metab Disord.* 2002, 26:633-9. A cohort study of 931 unrelated Caucasian females were genotypde for the Gly389Arg (rs1800861) variant in ADRB1. Each ARG allele was associated with a higher body weight (2.91kg, p=0.01) and BMI (0.86kg/m^2^, p=0.03).

**rs2227564 PLAU**

1) Bégin P, Tremblay K, Daley D, Lemire M, Claveau S, Salesse C, Kacel S, Montpetit A, Becker A, Chan-Yeung M, Kozyrskyj AL, Hudson TJ, Laprise C. **Association of urokinase-type plasminogen activator with asthma and atopy.** *Am J Respir Crit Care Med.* 2007, 175:1109-16. This is a study of 231 asthmatic French-Canadian families (1,139 subjects). The PLAU allele (rs2227564 – C allele) was associated with asthma (p= 0.011) using a dominant model of parental transmission.

2) Riemenschneider M, Konta L, Friedrich P, Schwarz S, Taddei K, Neff F, Padovani A, Kölsch H, Laws SM, Klopp N, Bickeböller H, Wagenpfeil S, Mueller JC, Rosenberger A, Diehl-Schmid J, Archetti S, Lautenschlager N, Borroni B, Müller U, Illig T, Heun R, Egensperger R, Schlegel J, Förstl H, Martins RN, Kurz A. **A functional polymorphism within plasminogen activator urokinase (PLAU) is associated with Alzheimer's disease** *Hum Mol Genet.* 2006, 15:2446-56. This is a case control study using four independent population samples for a total of 1751 individuals. The PLAU SNP rs2227564 was associated with Alzheimer’s disease in all four populations (p=0.001 to 0.03).

**rs1799750 MMP1**

1) Dörr S, Lechtenböhmer N, Rau R, Herborn G, Wagner U, Müller-Myhsok B, Hansmann I, Keyszer G. **Association of a specific haplotype across the genes MMP1 and MMP3 with radiographic joint destruction in rheumatoid arthritis.** *Arthritis Res Ther.* 2004, 6:R199-207. A case control study of 308 rheumatoid authritis patients and 110 healthy Caucasian controls. Patients homozygous for the 2G promoter allele (rs1799750) were at increased risk of radiographic damage in the first 15 years of disease onset (OR 2.41. p=0.012).

2) Joos L, He JQ, Shepherdson MB, Connett JE, Anthonisen NR, Paré PD, Sandford AJ. **The role of matrix metalloproteinase polymorphisms in the rate of decline in lung function.** *Hum Mol Genet*. 2002, 11:569-76. This is a cohort study of the 3216 (595 Caucasian) persistent smokers in the NHLBI, lung health study. The -1607 GG allele was associated with a fast rate of lung function decline in Caucasian patients (OR1.61, p=0.02).

**rs1063856 VWF**

1) Lacquemant C, Gaucher C, Delorme C, Chatellier G, Gallois Y, Rodier M, Passa P, Balkau B, Mazurier C, Marre M, Froguel P. **Association between high von willebrand factor levels and the Thr789Ala vWF gene polymorphism but not with nephropathy in type I diabetes. The GENEDIAB Study Group and the DESIR Study Group*.*** *Kidney Int.* 2000, 57:1437-43. This is a cohort study of 493 type I diabetic subjects with proliferative retinopathy. The Thr789Ala (rs1063856) allele of the vWF gene was significantly associated with coronary heart disease (p=0.002) but not with nephropathy in this patient population.

2) Lee YS, Miller M, Hassett AC, Lee-Kim BY, Kim JJ, Haracznak M, Ranade S, Wood J, Reddy R, Kupfer DJ, Bontempo F, Nimgaonkar VL .**von Willebrand's disease and psychotic disorders: co-segregation and genetic associations**. *Bipolar Disord.* 2004, 6:150-5. This study is case-control study of 194 patents with psychiatric disorders and their parents with 183 unrelated controls. There was increased transmission of the A allele of vWF allele 2365A>G (rs1063865) in patients with bipolar disorder type I and this allele was significantly associated with disease (p=0.04).

**rs6313 HTR2A**

1) Abdolmaleky HM, Faraone SV, Glatt SJ, Tsuang MT. **Meta-analysis of association between the T102C polymorphism of the 5HT2a receptor gene and schizophrenia.** *Schizophr Res.* 2004, 67:53-62. This is a meta-analysis including 31 case control and 5 family studies found that the C allele of T102C (rs6313) was significantly associated with schizophrenia combined OR in the family studies of 1.3.

2) Jokela M, Keltikangas-Järvinen L, Kivimäki M, Puttonen S, Elovainio M, Rontu R, Lehtimäki T. **Serotonin receptor 2A gene and the influence of childhood maternal nurturance on adulthood depressive symptoms***. Arch Gen Psychiatry*. 2007, 64:356-60. This study was a cohort study of a subset of 1212 Finnish males. Individuals that experienced high maternal nurturance in childhood experienced lower depressive scores if they carried the T allele of the HTR2a gene T102C polymorphism (rs6313).

**rs2236225 MTHFD1**

1) Kempisty B, Sikora J, Lianeri M, Szczepankiewicz A, Czerski P, Hauser J, Jagodzinski PP. **MTHFD 1958G>A and MTR 2756A>G polymorphisms are associated with bipolar disorder and schizophrenia.** *Psychiatr Genet.* 2007, 17:177-81. In a case control study of 22 bipolar 200 schizophrenic and 300 controls, MTHFD1 1958G>A (R653Q, rs2236225) carriers of the A allele were at an increased risk for the development of bipolar disorder type I or schizophrenia (OR 1.74 p=0.0027 for a single allele and OR2.667 p=0.002 for two alleles).

2) Brody LC, Conley M, Cox C, Kirke PN, McKeever MP, Mills JL, Molloy AM, O'Leary VB, Parle-McDermott A, Scott JM, Swanson DA. **A polymorphism, R653Q, in the trifunctional enzyme methylenetetrahydrofolate dehydrogenase/methenyltetrahydrofolate cyclohydrolase/formyltetrahydrofolate synthetase is a maternal genetic risk factor for neural tube defects: report of the Birth Defects Research Group.** *Am J Hum Genet.* 2002, 71:1207-15. The study consisted of 319 triads with an individual affected by neural tube defects and a control population of 699 individuals who did not give birth to a child with NTD. The Q allele of the MTHFD1 R653Q increased the risk of neural tube defects (OR 1.2 p=0.025 for a single allele and 1.52 p=0.003 for carriers of two alleles).

**rs1800588 LIPC**

1) Isaacs A, Sayed-Tabatabaei FA, Njajou OT, Witteman JC, van Duijn CM. **The -514 C->T hepatic lipase promoter region polymorphism and plasma lipids: a meta-analysis.** *J Clin Endocrinol Metab.* 2004, 89:3858-63. This study is a meta-analysis of 25 studies totaling in more than 24,000 individuals. Carriers of at least one T allele of -514C/T (rs1800588) had an increased HDL level (0.04 mmol/liter, p=<0.001) and this increase doubled in homozygous carriers (0.09mmol/liter p<0.001).

2) Hokanson JE, Kamboh MI, Scarboro S, Eckel RH, Hamman RF. **Effects of the hepatic lipase gene and physical activity on coronary heart disease risk.** *Am J Epidemiol.* 2003, 158:836-43. This study is a cohort study of 966 individuals consisting of both Hispanic and non-Hispanic Whites. Individuals homozygous for the T allele (-480C>T, rs1800588) were at increased risk of coronary heart disease if they had sedentary or moderate levels of physical activity (hazard ratio =2.58, p=0.003).

**rs243865 MMP2**

1) Rodriguez-Lopez J, Perez-Pampin E, Gomez-Reino JJ, Gonzalez A. **Regulatory polymorphisms in extracellular matrix protease genes and susceptibility to rheumatoid arthritis: a case-control study.** *Arthritis Res Ther.* 2006, 8:R1. A case control study of 550 rheumatoid arthritis patients and 652 controls of Spanish decent. The T allele of MMP2 -1306C/T (rs243865) conferred an increased risk of rheumatoid arthritis (OR 1.27, p= 0.013). This association was increased when other gene-gene interactions were added.

2) O-Charoenrat P, Khantapura P**. The role of genetic polymorphisms in the promoters of the matrix metalloproteinase-2 and tissue inhibitor of metalloproteinase-2 genes in head and neck cancer.** *Oral Oncol.* 2006, 42:257-67. This study is a case control study of 239 head and neck cancer patients and 250 frequency matched controls in an ethnic Thai population. The MMP2 CC allele (-1306 C/T, rs243865) was associated with an increased risk of cancer (OR 1.97), and adverse clinicopathological variables.

**rs4673 CYBA**

1) Perianayagam MC, Liangos O, Kolyada AY, Wald R, MacKinnon RW, Li L, Rao M, Balakrishnan VS, Bonventre JV, Pereira BJ, Jaber BL. **NADPH oxidase p22phox and catalase gene variants are associated with biomarkers of oxidative stress and adverse outcomes in acute renal failure.** *J Am Soc Nephrol*. 2007, 18:255-63. This study is a prospective cohort of 200 hospitalized patients with established acute renal failure. The presence of a T-allele (+242C/T, rs4673) was associated with a 2.1 fold higher odds for dialysis requirement or hospital death (p=0.01).

2) Fan M, Kähönen M, Rontu R, Lehtinen R, Viik J, Niemi M, Nieminen T, Niemelä K, Pörsti I, Kööbi T, Turjanmaa V, Lehtimäki T. **The p22phox C242T gene polymorphism is associated with a reduced risk of angiographically verified coronary artery disease in a high-risk Finnish Caucasian population. The Finnish Cardiovascular Study.** *Am Heart J.* 2006, 152:538-42. A case control study of 402 high risk Finnish Caucasion patients. The T allele (+242C/T, rs4673) of CYBA was protective against the development of coronary artery disease (OR 0.531, p=0.009) despite exposure to significant coronary risk factors.

**rs708272 CETP**

1) Porchay-Baldérelli I, Péan F, Bellili N, Jaziri R, Marre M, Fumeron F; DIABHYCAR Study Group. **The CETP TaqIB polymorphism is associated with the risk of sudden death in type 2 diabetic patients.** *Diabetes Care*. 2007, 30:2863-7. A cohort study of 3124 French individuals with type 2 diabetes. Individuals homozygous for the CETP TaqIB allele (rs708272) B1 allele were at increased risk of coronary heart disease and sudden death (Hazard ratio 1.51 p=0.02) than carriers of at least one B2 allele.

2) Borggreve SE, Hillege HL, Wolffenbuttel BH, de Jong PE, Zuurman MW, van der Steege G, van Tol A, Dullaart RP; PREVEND Study Group. **An increased coronary risk is paradoxically associated with common cholesteryl ester transfer protein gene variations that relate to higher high-density lipoprotein cholesterol: a population-based study**. *J Clin Endocrinol Metab.* 2006, 91:3382-8. This study is a population based study of 8141 predominantly Caucasian individuals . Carriers of the CETPTaqIB (rs708272) B2 allele were at increased risk of coronary artery disease (Adjusted OR 1.62 p<0.01)

**rs1800012 COL1A1**

1) Mann V, Ralston SH. **Meta-analysis of COL1A1 Sp1 polymorphism in relation to bone mineral density and osteoporotic fracture**.*Bone.* 2003, 32:711-7. This study is a meta-anaylysis of 26 published studies with 7849 total participants. The s allele of Col1a1 Sp polymorphism (rs1800012) increased the risk of fracture (OR1.26 p=0.002 for heterozygotes and OR 1.78, p=0.0003 for homozygotes).

2) Speer G, Szenthe P, Kósa JP, Tabák AG, Folhoffer A, Fuszek P, Cseh K, Lakatos P. **Myocardial infarction is associated with Spl binding site polymorphism of collagen type 1A1 gene.** *Acta Cardiol*. 2006, 61:321-5. This is a case-control study of 136 patients with myocardial infartion and 212 age-matched control subjects. Patients with at least one s allele of the Col1A1 Sp1 polymorphism (rs1800012) experienced poorer survival than those with the SS allele and had more cardiovascular risk factors.

**rs4291 ACE**

1) Baghai TC, Binder EB, Schule C, Salyakina D, Eser D, Lucae S, Zwanzger P, Haberger C, Zill P, Ising M, Deiml T, Uhr M, Illig T, Wichmann HE, Modell S, Nothdurfter C, Holsboer F, Müller-Myhsok B, Möller HJ, Rupprecht R, Bondy B. **Polymorphisms in the angiotensin-converting enzyme gene are associated with unipolar depression, ACE activity and hypercortisolism**. *Mol Psychiatry*. 2006, 11:1003-15. This is a case control study of two independent case control studies for a total of 243 unrelated unipolar depressed patients and 1479 healthy controls. ACE polymorphism rs4291 was significantly associated with unipolar major depression.

2) Kehoe PG, Katzov H, Andreasen N, Gatz M, Wilcock GK, Cairns NJ, Palmgren J, de Faire U, Brookes AJ, Pedersen NL, Blennow K, Prince JA. **Common variants of ACE contribute to variable age-at-onset of Alzheimer's disease.** *Hum Genet*. 2004, 114:478-83. A cohort study of 2861 individuals from three European populations. ACE promoter polymorphism rs4291 was independently associated with age of onset of Alzheimer’s disease (p=0.0095).

**rs4792311 ELAC2**

1) Noonan-Wheeler FC, Wu W, Roehl KA, Klim A, Haugen J, Suarez BK, Kibel AS. **Association of hereditary prostate cancer gene polymorphic variants with sporadic aggressive prostate carcinoma.** *Prostate.* 2006, 66:49-56. A case control study of European-American males with metastatic prostate cancer. The carriers of at least one L allele of the ELAC2 S217L polymorphism (rs4792311), were at an increased risk of prostate cancer (OR =1.54) and when combined with an RNASEL allele this risk increased (OR2.66).

2) Stanford JL, Sabacan LP, Noonan EA, Iwasaki L, Shu J, Feng Z, Ostrander EA. **Association of HPC2/ELAC2 polymorphisms with risk of prostate cancer in a population-based study.** *Cancer Epidemiol Biomarkers Prev.* 2003, 12:876-81. A case-control study of 591 middle aged men with prostate cancer and 538 controls from the King County, Washington, USA area. Men with at least one L allele of ELAC2 S217L were at increased risk of prostat cancer (OR 1.34) with a stronger association for homozygous L alleles (OR 1.73).

**rs16430 ENOSF1/TYMS**

1) Shi Q, Zhang Z, Neumann AS, Li G, Spitz MR, Wei Q. **Case-control analysis of thymidylate synthase polymorphisms and risk of lung cancer.** *Carcinogenesis.* 2005, 26:649-56. This is a case control study of 1055 non-Hispanic white lung cancer patients and 1140 cancer-free controls. The TS3’UTR 6bp deletion allele was associated with an increased risk of lung cancer. Individuals with at least one 6bp insertion were at increased risk of lung cancer (OR=1.52) this risk was increased when the individuals were greater when at least one of the following variables was added. over the age of 55, male, current heavy smokers, and current drinkers. With the highest OR occurring in current drinkers with the allele (OR=3.17).

2) Zhang Z, Shi Q, Sturgis EM, Spitz MR, Hong WK, Wei Q. **Thymidylate synthase 5'- and 3'-untranslated region polymorphisms associated with risk and progression of squamous cell carcinoma of the head and neck.** *Clin Cancer Res*. 2004, 10:7903-10. A case control study of 704 non Hispanic White individuals with squamous cell carcinoma of the head and neck and 1,085 unrelated control individuals. the 0bp/0bp genotype of the TS3’UTR (rs16430) was associated with a decreased risk of head and neck cancer (OR=0.67).

**rs601338 FUT2**

1) Kindberg E, Hejdeman B, Bratt G, Wahren B, Lindblom B, Hinkula J, Svensson L. **A nonsense mutation (428G-->A) in the fucosyltransferase FUT2 gene affects the progression of HIV-1 infection.** *AIDS.* 2006, 20:685-9. This study is a case control study in the Swedish population with 15 HIV infected long term non-progressors and 19 HIV infected progressors and 276 Swedish blood donors. The FUT2 G>A (rs601338) A/A allele was significantly over represented among long term non-progressors.

2) Larsson MM, Rydell GE, Grahn A, Rodriguez-Diaz J, Akerlind B, Hutson AM, Estes MK, Larson G, Svensson L. **Antibody prevalence and titer to norovirus (genogroup II) correlate with secretor (FUT2) but not with ABO phenotype or Lewis (FUT3) genotype.** *J Infect Dis.* 2006, 194:1422-7. This study is a cohort study of 105 Swedish blood donors. The donors were genotyped for secretor (FUT2, rs601338) phenotype. The results showed that nonsecretors had significantly lower antibody titers than did secretors (p<0.0001) and were more oftern antibody negative (p<0.05).

3) Azevedo M, Eriksson S, Mendes N, Serpa J, Figueiredo C, Resende L, Ruvoën-Clouet N, Haas R, Borén T, Le Pendu J, David L. **Infection by Helicobacter pylori expressing the BabA adhesin is influenced by the secretor phenotype**. *J Pathol.* 2008, 215:308-16. A cohort study of 304 *Helicobacter pylori* (Hp) infected individuals from Northern Portugal. Hp adherence was higher in wild-type or heterozygous individuals for FUT2 G428A (rs601338) (p<0.0001).

**rs688 LDLR**

1) Zou F, Gopalraj RK, Lok J, Zhu H, Ling IF, Simpson JF, Tucker HM, Kelly JF, Younkin SG, Dickson DW, Petersen RC, Graff-Radford NR, Bennett DA, Crook JE, Younkin SG, Estus S. **Sex-dependent association of a common low-density lipoprotein receptor polymorphism with RNA splicing efficiency in the brain and Alzheimer's disease.** *Hum Mol Genet.* 2008, 17:929-35. This study was a compilation of 3 case control studies for a total of 1457 men and 3055 women. The rs688 (T/T) allele was associated with increase risk of Alzheimer’s disease (OR1.49) in males.

2) Zhu H, Tucker HM, Grear KE, Simpson JF, Manning AK, Cupples LA, Estus S. **A common polymorphism decreases low-density lipoprotein receptor exon 12 splicing efficiency and associates with increased cholesterol.** *Hum Mol Genet.* 2007, 16:1765-72. The Framingham Offspring Study was used in this longitudinal cohort-study this consisted of 1314 individuals. The LDLR polymorphism rs688 T allele was associated with increased total cholesterol in women this was most evident in pre-menopausal women.

**rs7121 GNAS**

1) Hahn S, Frey UH, Siffert W, Tan S, Mann K, Janssen OE. **The CC genotype of the GNAS T393C polymorphism is associated with obesity and insulin resistance in women with polycystic ovary syndrome*.*** *Eur J Endocrinol.* 2006, 155:763-70. This is a case control study of 278 German women with poly cystic ovary syndrome and 820 controls. The C allele of the GNAS1 polymorphism T393C (rs7121) was associated with a higher mean body weight, a higher BMI and a higher indices of insulin resistance compared to women without a C allele.

2) Otterbach F, Callies R, Frey UH, Schmitz KJ, Wreczycki C, Kimmig R, Siffert W, Schmid KW. **The T393C polymorphism in the gene GNAS1 of G protein is associated with survival of patients with invasive breast carcinoma.** *Breast Cancer Res Treat.* 2007, 105:311-7. This is a cohort study of 279 patients with invasive breast cancer. Carriers of a single T allele of the T393C polymorphism had a less favorable course of the disease and the TT carriers were associated with decreased overall survival in multivariate analysis.

3) Oterino A, Ruiz-Alegría C, Castillo J, Valle N, Bravo Y, Cayón A, Alonso A, Tejera P, Ruiz-Lavilla N, Muñoz P, Pascual J. **GNAS1 T393C polymorphism is associated with migraine.** *Cephalalgia.* 2007, 27:429-34. This is a case-control study of 365 migraine patients and 347 healthy controls. Individuals homozygous for the C allele of the T393C polymorphism (rs7121) were at an increased risk for migraine (OR1.79, p=0.001).

**rs234706 CBS**

1) Boyles AL, Wilcox AJ, Taylor JA, Meyer K, Fredriksen A, Ueland PM, Drevon CA, Vollset SE, Lie RT. **Folate and one-carbon metabolism gene polymorphisms and their associations with oral facial clefts.** *Am J Med Genet A.* 2008 146A:440-9. This is a population based study of 326 Norwegian families with cleft lip with or without cleft palate (CL/P) and 191 families with cleft palate only (CLO). There was a reduced risk of CL/P when mothers carried at least one T allele of C699T (rs234706) (RR=0.94) this risk was further decreased when the mothers were homozygous for T (RR=0.50, p=0.008).

2) Urreizti R, Asteggiano C, Vilaseca MA, Corbella E, Pintó X, Grinberg D, Balcells S. **A CBS haplotype and a polymorphism at the MSR gene are associated with cardiovascular disease in a Spanish case-control study**. *Clin Biochem*. 2007, 40:864-8. This is a case control study of 140 Spanish coronary artery disease patients and 113 controls. The CBS haplotype (c.699C (rs234706)-c.844wt-c.1080C) was over-represented in coronary artery disease (OR 2.16).

**rs4680 COMT**

1) Annerbrink K, Westberg L, Nilsson S, Rosmond R, Holm G, Eriksson E. **Catechol O-methyltransferase val158-met polymorphism is associated with abdominal obesity and blood pressure in men.** *Metabolism.* 2008, 57:708-11. This study is a cohort study of 240 Swedish men. The COMT val158met allele (rs4680) was significantly associated with waist-to-hip ratio (p=0.002) and abdominal sagittal diameter (0.05) and systolic blood pressure (0.003). While WHR and blood pressure are correlated, when WHR and systolic blood pressure were regressed against one another they still remained significant indicating that COMT (rs4680) was independently associated with each factor.

2) Muntjewerff JW, Gellekink H, den Heijer M, Hoogendoorn ML, Kahn RS, Sinke RJ, Blom HJ. **Polymorphisms in catechol-O-methyltransferase and methylenetetrahydrofolate reductase in relation to the risk of schizophrenia.** *Eur Neuropsychopharmacol.* 2008, 18:99-106. In a study of 252 Dutch schizophrenia patients and 405 controls the COMT 324G>A polymorphism (rs4680) homozygous AA was associated with schizophrenia when in joint occurrence with MTHFR677TT genotype (OR=3.08 p=0.035).
